# Are antidepressants addictive? a literature review

**DOI:** 10.1192/j.eurpsy.2023.1399

**Published:** 2023-07-19

**Authors:** M. Turki, O. Elleuch, F. Sahnoun, H. E. Mhiri, A. Guermazi, S. Ellouze, N. Halouani, J. Aloulou

**Affiliations:** Psychiatry B, Hedi Chaker University Hospital of Sfax, Sfax, Tunisia

## Abstract

**Introduction:**

Nowadays, the rates of antidepressant prescription are high and increasing. In this context, the issue of whether these medications are addictive has been increasingly discussed.

**Objectives:**

The aim of this review was to explore the debate about the addictive property of antidepressants.

**Methods:**

We conducted a literature review in the Pubmed database, using the search terms “antidepressants”, “SSRI”, “tricyclic”, “addiction”, “dependence” in various combinations, and narrowing the search to the last 10 years, to identify articles about the addiction to antidepressants.

**Results:**

All the articles included in our study highlighted the fact that antidepressants were associated with withdrawal symptoms. These symptoms are heterogeneous, and long-lasting in some cases, and Paroxetine was shown to have particularly high rates of withdrawal symptoms.

Some articles reported a psychological and physical dependence on antidepressants. However, the most recent studies agreed that, using established classification systems and concepts and after integrating neurobiological and behavioral criteria, antidepressants are shown to have no addictive property.

**Conclusions:**

Antidepressants are proven to be associated with withdrawal symptoms. Whether or not these symptoms are enough to constitute an “addiction” remains controversial, as recent studies agree that antidepressants should not be classified as addictive substances.

**Disclosure of Interest:**

None Declared

